# Actions and feelings in sync: exploring the relationship between synchrony and empathy in children’s dyadic musical interactions

**DOI:** 10.3389/fpsyg.2025.1467767

**Published:** 2025-04-25

**Authors:** Persefoni Tzanaki, Tuomas Eerola, Renee Timmers

**Affiliations:** ^1^Department of Music, University of Sheffield, Sheffield, United Kingdom; ^2^Department of Music, Durham University, Durham, United Kingdom

**Keywords:** bidirectional relationship, interpersonal synchrony, trait empathy, induced empathy, state empathy, musical development, social bonding

## Abstract

**Introduction:**

This study investigated the relationship between interpersonal synchrony and empathy in children’s music-making. Drawing from a theoretical framework that suggests a bidirectional relationship between synchrony and empathy, the study examined three key aspects of this relationship: (1) the role of children’s trait empathy in achieving interpersonal synchrony; (2) synchrony’s effects on empathy following brief musical interactions; and (3) the role of experimentally induced empathy in moderating the social bonding effects of synchrony.

**Methods:**

Seventy-two pairs of primary school children participated in two experiments. The first involved free tapping, where participants were instructed to synchronize with one another. In the second experiment, synchrony was manipulated, using an apparatus that either facilitated or disrupted synchrony within pairs. Prior to this task, half of the pairs received a false message about their partner, intended to induce empathy. Trait empathy and social bonding were assessed via self-reported questionnaires.

**Results:**

Findings revealed that cognitive and affective trait empathy related to children’s ability to synchronize with one another, particularly when participants’ temporal performance was unstable (Aspect 1). In addition, brief synchronous musical interactions were found to promote empathy within pairs (Aspect 2). Our method to experimentally induce empathy was not sufficient to influence the social bonding effects of synchrony (Aspect 3). However, trait empathy, pairs’ gender composition and familiarity between children emerged as factors affecting the attainment of synchrony and the bonding experience of music-making.

**Discussion:**

This is the first empirical study investigating multiple aspects of the interplay between synchronizing and empathizing in children, paving the way for future exploration of the mechanisms allowing for a bidirectional relationship. The study outcomes can inform musical interventions leveraging this relationship to nurture children’s simultaneous musical and social development.

## 1 Introduction

In recent decades, empirical research has highlighted the advantages of musical interactions in shaping children’s socio-emotional development and relationships. An integral component of such interactions, interpersonal synchrony - defined as the temporal alignment of movements between individuals - can positively influence social closeness, perceived similarity and prosociality among primary pupils and toddlers (Rabinowitch and Knafo-Noam, 2015; Cirelli et al., 2014; Rabinowitch, 2023). Further supporting this, Rabinowitch et al. (2013) demonstrated a decade ago that long-term engagement in musical interactions involving interpersonal synchrony (hereafter also referred to as “synchrony”) can contribute to the development of affective empathy in children, a crucial skill for social interactions. This finding supports the role of synchrony in fostering emotional alignment, thereby strengthening social connections and empathy among children (Cross et al., 2012; Tzanaki, 2022; Rabinowitch, 2023).

While these studies suggest that synchrony can promote empathy from an early age, research in adults has indicated that this influence may operate in both directions, with empathy also contributing to synchrony. Specifically, Novembre et al. (2019) observed individuals with higher empathic perspective-taking skills synchronizing better with others than those with lower empathy, suggesting that empathic capacities facilitate internal simulation and prediction of others’ temporal behavior. This insight was further reinforced by Bamford and Davidson (2019), who found that those with high empathy are better at re-aligning their movements to music changing unexpectedly. This implies that brain areas responsible for empathizing might also be involved in perceiving and understanding temporal changes through music (Overy and Molnar-Szakacs, 2009), thereby supporting synchrony with others.

Further to these findings, recent studies have revealed an additional dimension of the synchrony-empathy relationship: the role of empathy in enhancing the social bonding effects of synchrony. Specifically, individuals with high empathy experience stronger bonding than those with low empathy when they observe or actively interact with virtual partners moving or tapping in synchrony with music (Stupacher et al., 2022; Tzanaki et al., 2024). These observations suggest that empathy may play a crucial role not only in achieving synchrony but also in experiencing the bonding effects of this temporal alignment.

This interplay between synchrony and empathy finds its roots in various parallels between the two phenomena. Empathy involves imagining and aligning with others’ emotional states (Singer and Lamm, 2009), while synchrony during music-making similarly requires predicting and adapting to others’ movements to produce a musically coherent outcome (Keller, 2014). Additionally, in studies beyond music, synchronizing with a partner’s movements in behavioral tasks has been shown to enhance the sharing of mental states, reinforcing the experience of empathy (Baimel et al., 2018; Koehne et al., 2016). These findings support the social alignment model by Shamay-Tsoory et al. (2019), which regards motor coordination and emotional and cognitive alignment in social interactions as processes influencing one another bidirectionally. Such reciprocal connections are evident in dance therapy, where techniques such as mirroring and synchrony enhance cognitive alignment among partners, fostering emotional connection and empathy (Behrends et al., 2012; Castro Jaramillo and Panhofer, 2021).

Drawing on such parallels, a theoretical model pertaining to musical interactions was developed by Tzanaki (2022), suggesting that during music-making, synchrony and empathy establish a positive feedback loop, reinforcing one another in a reciprocal manner. It has been hypothesized that musical partners utilize their empathic skills to predict and synchronize with their others’ temporal movements (Novembre et al., 2019), while their attained synchrony enhances perceived similarity and affiliation, thereby fostering metalizing and empathy (Baimel et al., 2018). This enhanced empathy, in turn, supports partners’ interpersonal synchrony (Novembre et al., 2019) and strengthens its subsequent social bonding effects (Tzanaki et al., 2024). The model bears significant implications, particularly for children, as musical interventions could harness this feedback loop to simultaneously enhance children’s musical and social skills and promote intergroup similarity (Tzanaki, 2022). Nonetheless, the framework is constructed on evidence primarily from studies in adults, with the implied bidirectional effects remaining largely theoretical. There is also limited research on the development of crucial unidirectional aspects of the feedback loop, necessitating further exploration before focusing on the bidirectional nature of this relationship and its implications.

In light of these research gaps, the present study investigated three unidirectional aspects of the feedback loop between empathy and synchrony, focusing on children’s interactions in a musical context. Across two experiments (Experiment 1: “Free synchrony task”; Experiment 2: “Manipulated synchrony task”), we tested three potential directions of influence between synchrony and empathy in pairs of primary school children. Specifically, we explored:

- Aspect 1: The role of empathy in facilitating synchrony between children.
- Aspect 2: The effects of synchrony on empathy following a brief musical interaction.
- Aspect 3: The contribution of empathy to the experience of social bonding following synchronous musical interactions with peers.

**Figure 1:**
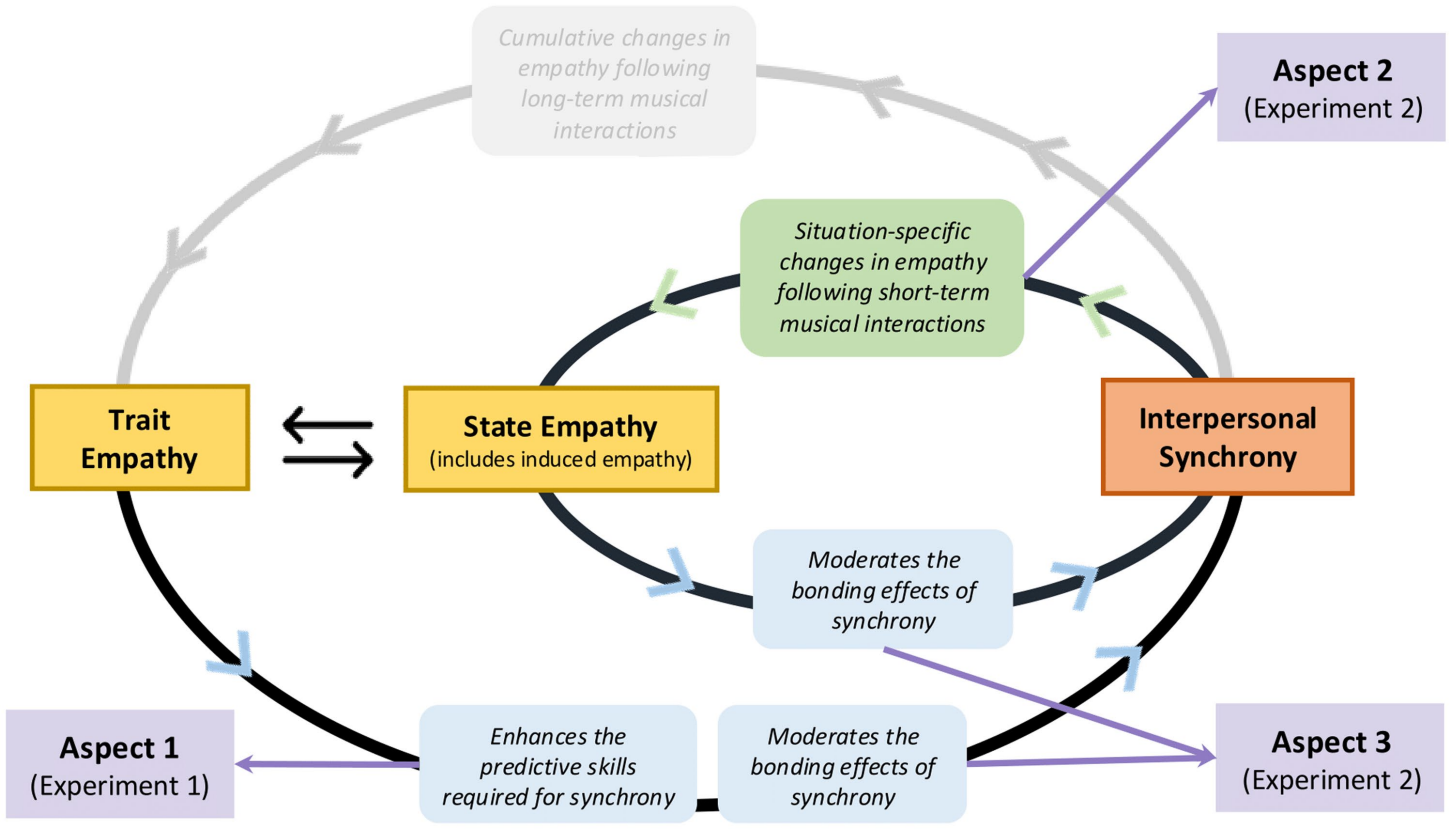
The positive feedback loop model (Tzanaki, 2022) adapted to highlight the aspects explored in the present study. The green and blue boxes match the colors of the arrows to indicate the direction of the effects investigated. The purple boxes and arrows indicate the three aspects explored in the present study. The grayed-out section is part of the model but was not investigated in this study.

These aspects aim to solidify the empirical basis of the feedback loop model (Tzanaki, 2022), paving the way for future research into the bidirectional nature of the synchrony-empathy relationship. Below, we outline how the aspects correspond to the experiments conducted. Given the multifaceted nature of empathy and synchrony, encompassing varying definitions across disciplines, we provide a glossary (Table 1) to clarify how these and other key terms are defined within this study. Additionally, Figure 1 presents an adapted version of the feedback loop model (Tzanaki, 2022), highlighting the aspects and research questions addressed here, with further details summarized in Table 2.

**Table 1:** Glossary of key terms used in this study.

Term	Definition in the present study
Interpersonal synchrony	Refers to the temporal alignment between participants’ strokes on the wooden claves within each pair. This was assessed by calculating the absolute asynchrony between participants’ onsets.
Trait empathy	The dispositional ability to identify and share others’ thoughts and emotional states. In this study, we captured via a self-reported questionnaire three dimensions of trait empathy: (1) Cognitive empathy, defined as the ability to understand another person’s thoughts and feelings; (2) Affective empathy, the capacity to share or feel another person’s emotional state; and (3) Somatic empathy, the bodily experience of emotions, such as a physical reaction to someone else’s distress.
State empathy	State empathy refers to how much one empathizes with others in a given situation. Individuals appraise a situation, with factors such as the environment and the people involved determining the level of empathy experienced. In Experiment 2, we asked participants to rate on a questionnaire how much they could understand the thoughts and feelings of their partners following their musical interactions.
Induced or experimentally manipulated empathy	Empathy can be experimentally induced, encouraging participants to imagine the emotional state of another person based on a fake scenario. In Experiment 2, half of the participants listened to a pre-recorded message, informing them about a fictious unfortunate situation involving their partner. This message was designed to induce empathy in these participants prior to their musical interactions.
Social bonding	In this study, social bonding was explored through three key aspects: (1) Social closeness, which refers to the perceived connection between individuals; (2) Perceived similarity, which reflects the extent to which individuals feel similar to one another in terms of attitudes, values, or behaviors; and (3) State empathy, which captures the momentary emotional understanding and sharing of another’s thoughts and feelings, as defined above. These three aspects offer a comprehensive approach to understanding social bonding. They have also been examined in previous studies, allowing for comparisons.
Musical interaction	Musical interactions in this study involved dyadic engagements with singing and rhythmic playing on percussion instruments.

**Table 2:** Aspects, research questions, and hypotheses of the present study.

Aspect investigated	Research questions	Hypotheses	Experiment
Aspect 1: The effects of trait empathy on children’s synchrony	(1) Does trait empathy facilitate synchrony in children’s dyadic musical interactions?	(1) Children with higher empathy will synchronize better with their partners than those with low empathy.	Experiment 1: “Free synchrony task”
	(2) Which empathic facet (cognitive, affective, somatic) contributes more to these effects?	(2) Cognitive and somatic empathy will contribute to children’s synchrony by supporting the internal simulation of partners’ actions and their subsequent temporal alignment (Iacoboni, 2009).	
	(3) What is the role of leadership? Do highly empathic children, performing as followers, synchronize better with their partners than those with lower empathy?	(3) Leadership assignments will interact with empathy, with highly empathic children synchronizing better when instructed to follow.	
Aspect 2: The effects of synchrony on children’s empathy and social bonding following brief musical interactions	(4) Does interpersonal synchrony enhance social bonding (closeness, perceived similarity and state empathy) following brief musical interactions?	(4) Interpersonal synchrony will enable children to bond with their partners (a) and exhibit state empathy (b).	Experiment 2: “Manipulated synchrony task”
	(5) What are the effects of interpersonal synchrony on children’s experience of state empathy following brief musical interactions?		
Aspect 3: The role of empathy (trait and induced) in children’s experience of social bonding following synchrony.	(6) Does trait empathy influence the social bonding effects of interpersonal synchrony in children?	(5) Children displaying higher empathy levels will experience stronger social bonding when synchronized with a partner. (6) Affective empathy will play a more significant role in these effects, considering its association with experiencing others’ emotional states.	Experiment 2: “Manipulated synchrony task”
	(7) Does induced (experimentally manipulated) empathy influence these social bonding effects of synchrony?	(7) Children in the induced empathy group will report higher social affiliation following interpersonal synchrony than those not exposed to the fictional story.	
	(8) Do the effects of induced empathy change depending on children’s trait empathy (is there an interaction between trait and induced empathy)?	(8) Children with higher levels of trait empathy will respond more strongly to the empathy manipulation message and experience stronger bonding with their synchronous partners.	

### 1.1 Aspect 1: the effects of trait empathy on children’s synchrony

Aspect 1 was investigated in Experiment 1 (“Free synchrony task”), replicating elements of Novembre et al.’s study (2019). The experiment focused on trait empathy and explored how it might facilitate children’s synchrony during dyadic musical interactions. This novel aspect, not previously investigated in young musical novices, aimed to shed light on children’s empathic skills and their involvement in predicting and adapting to partners’ temporal behavior. Similarly to the original study, the experiment also explored the effects of leadership, assessing how leading or following a musical interaction impacts children’s alignment with a partner.

Our methodology differed from that of Novembre et al. (2019), who used music boxes that rotated with a handle for participants to synchronize. Instead, we asked pairs of children to sing together and play rhythmically on percussion instruments, resembling traditional musical activities. Moreover, while Novembre et al.’s study focused solely on empathic perspective-taking (often overlapping with cognitive empathy), here we extended the exploration to affective and somatic empathy (Blair, 2005). Affective empathy involves the sharing of others’ emotional states and has been argued to be an essential element of musical interactions, cultivating strong social connections (Cross et al., 2012). Somatic empathy relates to the automatic bodily responses to one’s actions or emotions (e.g., spontaneously smiling when seeing someone laughing; Raine and Chen, 2018) and is considered a prerequisite for cognitive and affective empathy (van der Graaff et al., 2016). This somatic manifestation holds additional significance for the present study, given the hypothesized involvement of motor simulations of others’ actions in both synchrony (Novembre et al., 2012) and empathy (Iacoboni, 2009). Taking a broader perspective on empathy sought to illuminate aspects that might be overlooked when focusing solely on cognitive empathy.

Building upon Novembre et al. (2019), we hypothesized that children with higher trait empathy would synchronize better with their partners compared to those with lower empathy (Hypothesis H1). Delving into each empathic facet, and in addition to the cognitive empathy effects observed by Novembre et al. (2019), we also anticipated somatic empathy to contribute to children’s synchrony (H2) by supporting the simulation of partners’ actions (Iacoboni, 2009). Lastly, we hypothesized that leadership assignments would interact with empathy, with highly empathic children synchronizing better when instructed to follow, as observed by Novembre et al. (2019, H3).

### 1.2 Aspect 2: the effects of synchrony on children’s empathy and social bonding following brief musical interactions

Aspect 2 focused on the reverse direction of the synchrony-empathy relationship, namely the effects of synchrony on children’s experience of empathy and social bonding. While Rabinowitch et al. (2013) observed changes in children’s trait empathy following a 9-month-long musical program, it remains unclear whether short-term musical engagements would exhibit similar effects. Prior research has indicated that brief interactions involving coordinated movements can encourage children’s closeness and perceived similarity (Rabinowitch and Knafo-Noam, 2015); however, such evidence stems from research outside the realm of music, leaving the effects of synchrony in short-term musical interactions unexplored. To address this gap, Experiment 2 (“Manipulated synchrony task”) investigated whether two-minute-long musical dyadic interactions would be adequate to foster empathy and social bonding in children.

Given the brief nature of such musical interactions, investigating changes in trait empathy (as in Rabinowitch et al., 2013) would be inappropriate. Instead, we explored the impact of synchrony on situational (also known as state) empathy, drawing from relevant studies in adults (e.g., Baimel et al., 2018; Koehne et al., 2016). This approach views empathy as a dynamic process, subject not only to dispositional manifestations but also to individuals’ appraisals of a given situation (Lamm et al., 2007). Therefore, we anticipated that short-term synchronous music-making would facilitate this appraisal (Tzanaki et al., 2024), enabling children to bond and empathize with their partners when synchronizing with them (H4).

### 1.3 Aspect 3: the role of empathy (trait and induced) in children’s experience of social bonding following synchrony

For the final aspect, we examined how individual differences in empathy might explain variations in the experience of social bonding following short-term musical interactions. Expanding on relevant research in adults (Stupacher et al., 2022; Tzanaki et al., 2024), we explored whether trait empathy (cognitive, affective and somatic) heightens children’s experience of social bonding and state empathy following synchronous musical interactions. We hypothesized that children displaying higher trait empathy would experience stronger social bonding and state empathy than those with low empathy when synchronizing with a partner (H5). In addition, affective empathy was expected to play a more significant role in these effects, considering its association with experiencing others’ emotional states (de Waal, 2007; H6).

Lastly, in addition to trait empathy, we examined how experimentally induced empathy might amplify the bonding effects of synchrony. Specifically, participants were exposed to a fictional story about their partners, inspired by van Lange (2008) and Miu and Baltes (2012), aiming to redirect attention to partners’ emotional states. This manipulation, not previously examined in this context, aimed to further illuminate the role of empathy in facilitating social bonding through synchrony. We hypothesized that children in the induced empathy group would report higher social affiliation following synchronous music-making than those not exposed to the fictional story (H7; Stupacher et al., 2022; Tzanaki et al., 2024). Furthermore, we anticipated an interaction between trait and induced empathy, with children with higher trait empathy responding more strongly to the fictional message, experiencing stronger bonding with their synchronous partners (H8).

To facilitate readability and understanding, the methods and results of each experiment are reported separately, while their outcomes are collectively discussed in the General Discussion section. Both experiments were approved by the Department of Music Ethics Committee at the University of Sheffield. Data collection was carried out in Greece, leveraging the first author’s teaching background in the country, which provided access to a broader network of schools. All materials and experimental procedures were administered in Greek. A preceding pilot study with eight bilingual (Greek and English) pupils from the Greek School of Sheffield was conducted to verify the appropriateness of the methodology chosen for the intended age group.

## 2 Methods for Experiment 1: “Free synchrony task”

### 2.1 Summary

During Experiment 1, pairs of participants sang and played wooden claves in synchrony with their song. Microphones attached to the claves recorded their performance, allowing for the assessment of pairs’ temporal alignment (interpersonal synchrony) across trials (H1 and H2). Additionally, leadership roles were assigned for some of the trials, allowing the exploration of H3. Trait empathy was assessed via a self-reported questionnaire prior to the experiment.

### 2.2 Participants

Pupils were recruited from five primary schools in Heraklion (Greece). An *a priori* power analysis in G*Power 3.1 (Faul et al., 2009) suggested that a sample size of 109 participants would be sufficient to detect medium effect sizes (f^2^ = 0.15) at a significance level (*α*) of 0.05 and 80% power (1-*β*). Initially, 164 children completed the study; however, after following the exclusion process described in Supplementary Appendix 1, the final sample size comprised 144 children (72 pairs). Parental/caregiver consent and assent from children were obtained prior to the experiment.

Participants’ ages ranged from 10 to 12 years (*M* = 11.04 years, *SD* = 0.73). This was selected based on research indicating that children at this age can adequately synchronize with rhythmic stimuli (Drake et al., 2000), have developed a level of empathy (Stietz et al., 2019), and are able to follow instructions. This age also aligned with previous studies, allowing for outcome comparisons (Rabinowitch et al., 2013; Rabinowitch and Knafo-Noam, 2015). Table 3 presents additional demographic information. Children were randomly allocated to pairs without controlling for gender. Approximately half of the pairs were same-gender (male–male or female–female), while the rest were mixed.

**Table 3:** Summary of participants’ characteristics derived from a demographics questionnaire.

Characteristic	Options	Experiment 1	Experiment 2
N	Participants / Pairs	144/72	138/69
Age		*M* = 11.04 years, *SD* = 0.73	*M* = 11.05 years, *SD* = 0.72
Gender	Female/Male participants	77 (53.5%)/67 (46.5%)	74 (53.6%)/64 (46.4%)
	N of mixed-gender pairs	37 pairs	36 pairs
	N of female–female pairs	20 pairs	19 pairs
	N of male–male pairs	15 pairs	14 pairs
Musical experience/training	No prior experience	70.1%	70.3%
	Less than a year/only at school*	17.4%	17.4%
	1–5 years of experience	9%	9.4%
	More than 5 years of experience	3.5%	2.9%
Familiarity within pairs (previous acquaintances)	Not knowing each other at all	35.4%	35.5%
	Knowing each other a little bit	38.1%	39.1%
	Knowing each other quite a bit	17.3%	16.6%
	Knowing each other well	5.5%	5%
	Knowing each other very well/ friends	3.4%	3.6%

M, Mean; SD, Standard Deviation. *Children in Greece tend to attend after-school music clubs, conservatoires or music lessons with private tutors. Music at primary schools often includes 45-min weekly theoretical lessons (e.g., the history of music) or musical games in groups. The practice of musical instruments at school is very rare. Some schools offer the option of joining a choir.

### 2.3 Questionnaires

A demographics questionnaire collected information about ages, school year, musical interests and prior musical experiences (Table 3). In order to assess participants’ trait empathy, we used the Cognitive, Affective and Somatic Empathy Scales (CASES) (Raine and Chen, 2018), measuring positive and negative dimensions of children’s empathy. Permission for translating and using CASES was granted by its first author (Prof. Adrian Raine) and the © 2018 Society of Clinical Child and Adolescent Psychology, Division 53, American Psychological Association. The questionnaire encompassed 30 statements describing everyday scenarios, with participants assessing how much the items reflected their experiences using a 3-point Likert scale (“*Rarely*,” “*Sometimes*,” “*Often*”). The original English version of CASES was translated into Greek and validated for the present study (Tzanaki et al., 2024; manuscript in preparation). The term “empathy” was not explicitly mentioned; instead, participants were told that the questionnaire explored their feelings in everyday situations. Participants with three or more missing responses or two gaps within the same subscale (cognitive, affective or somatic) were excluded from the analysis. All other single missing values were replaced with the mean of ratings provided within that particular subscale. A total score for each subscale and an overall empathy score were computed for each participant.

The experiment was completed in pairs randomly formed with pupils from different classrooms within the same school to ensure minimal prior social interactions. However, to further determine the extent of familiarity within pairs, we asked participants to indicate this individually on a 5-point Likert scale, ranging from 0 (“*We do not know each other at all”*) to 5 (*“We know each other well and are good friends”*).

### 2.4 Stimuli and equipment

The experiment involved pairs of participants singing the Greek version of *“Twinkle, Twinkle, Little Star”* while rhythmically performing on wooden claves. The song was chosen due to its widespread familiarity and simple rhythmical structure. Wooden claves with attached contact microphones (OTraki AD-35) were used. Participants’ strokes were recorded on Steinberg Cubase 11 via a Steinberg UR22 MKII audio interface connected to an HP Spectre x360 laptop. The recordings were exported as audio files (.wav) for analysis.

Participants also undertook a baseline task, performing in synchrony with a five-bar steady metronome using the wooden claves. After three bars, the metronome gradually diminished in volume over two bars while participants maintained the tempo for three additional bars. Their final eight-bar performance was recorded on Audacity (3.2.1). The metronome was set to 120 beats per minute (bpm), a comfortable tempo for rhythmical performance within this age group (Drake et al., 2000).

### 2.5 Experimental procedure

**Figure 2:**
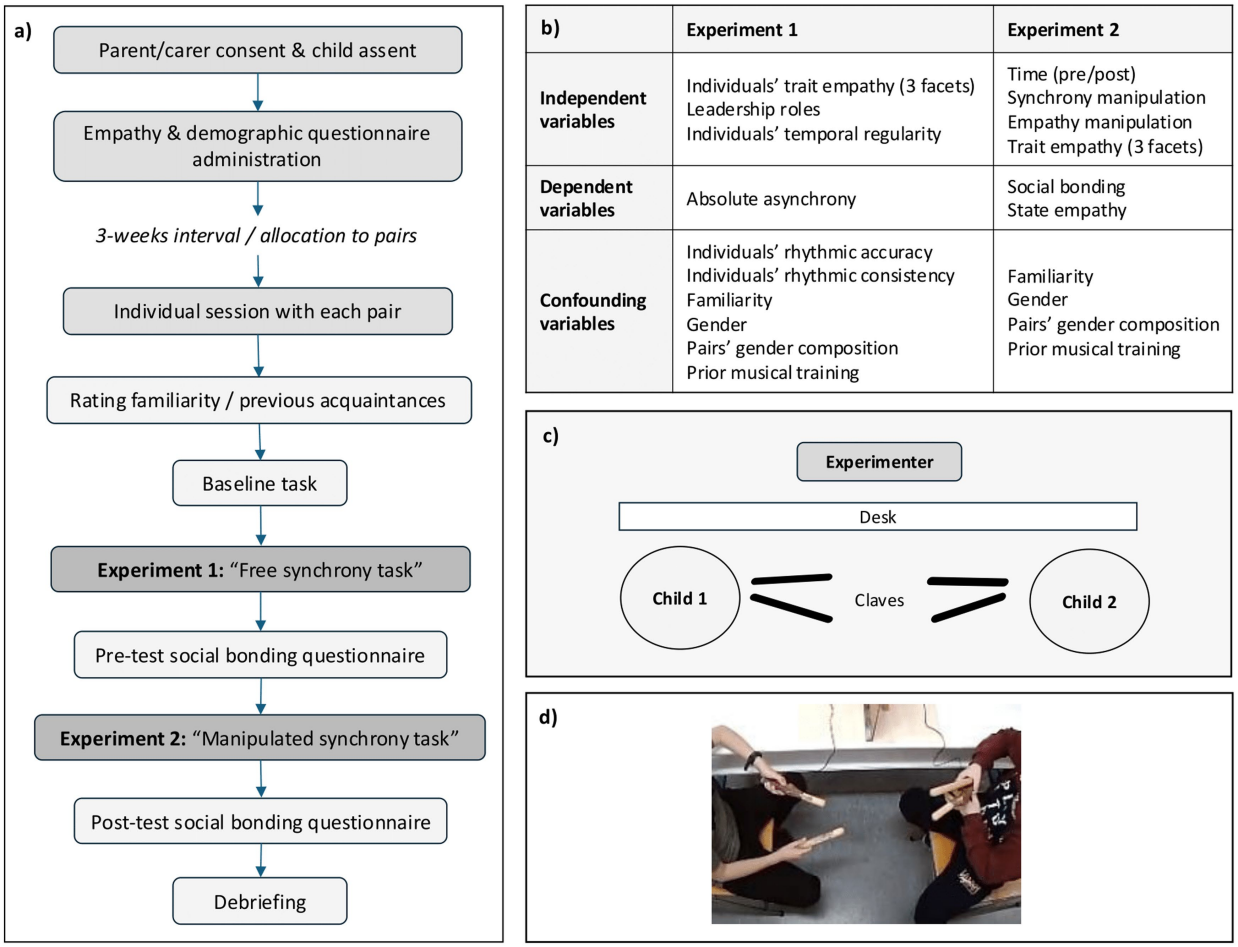
**(a)** The experimental procedure. **(b)** All variables related to the two experiments. **(c)** A panoramic perspective of the room setup. Participants were encouraged to look at each other during their musical interactions. Claves were used in Experiment 1 and replaced with other percussion instruments for Experiment 2. **(d)** Screenshot from a video recording of one of the sessions in Experiment 1.

Figure 2 illustrates the experimental procedure. The study commenced with the experimenter (first author) administering in a whole-class setting the demographics and empathy questionnaires. The experimenter remained present during completion, providing assistance where required. Pairs of children from different classrooms within the same school were randomly formed.

Three weeks later, Experiment 1 was conducted separately for each pair in a quiet room within their school. Figure 2 presents the room setup. Each session started with participants rating how well they knew each other before completing the baseline task individually. The baseline assessment evaluated children’s rhythmic accuracy and consistency, informing the study outcomes about potential confounding effects of individuals’ challenges in sensorimotor synchronization.

**Table 4:** The final best-fitting model of absolute asynchrony and its parameter estimates.

Model			Random effects			AIC	BIC
Pairs’ absolute asynchrony ~ Individuals’ total empathy * Individuals’ temporal regularity + Pairs’ gender composition			(1 | Pair: Participant)			8210.75	8251.75
Fixed effects	*β*	*SE*	*df*	*t*	*p*	*η_p_^2^*	95% CI
**(intercept)**	**53.83**	**5.05**	**627.37**	**10.65**	<0.001***	**-**	**[43.93, 63.74]**
**Individuals’ total empathy**	**−0.30**	**0.11**	**634.46**	**−2.57**	0.010*	**0.01**	**[−0.54, −0.07]**
**Individuals’ temporal regularity**	**−40.33**	**9.46**	**1232.68**	**−4.26**	<0.001***	**0.01**	**[−58.87, −21.78]**
Ind. tot. Emp. * Ind. temp. Regul.	**0.59**	**0.22**	**1229.50**	**2.64**	0.008**	**0.004**	**[0.15, 1.03]**
**Female–Female pairs (F-F)**	**−3.37**	**1.13**	**133.32**	**−2.96**	0.003**	**0.10**	**[−5.61–1.14]**
Male–Male pairs (M-M)	1.63	1.28	132.97	1.26	0.208	0.10	[−0.89, 4.15]

The variance explained by the random effect was low; however, it was kept in the model as it was meaningful given the experimental design. For “pairs’ gender composition,” RStudio compared Female–Female and Male–Male pairs with mixed-gender pairs. AIC, Akaike Information Criterion; BIC, Bayesian Information Criterion; *β*, Coefficient estimate; *SE*, Standard Error; *df*, Degrees of freedom. *t*-values and *p*-values associated with *β. η_p_*^2^, Partial eta-squared measuring effect size: Small = 0.01; Medium = 0.06; Large = 0.14. CI, Confidence Intervals. Significance levels: ***p < 0.001, **p < 0.01, *p < 0.05.

Subsequently, participants practiced singing the Greek “Twinkle Twinkle Little Star” with the experimenter assisting by singing parts of the song and displaying the lyrics on paper. Following this, pairs were instructed to imagine being part of a music band and sing together while playing on the beat using their wooden claves. To ensure participants would perform in synchrony, they were instructed to “copy” each other, avoiding the term “synchronize,” considered as not age appropriate. Children’s singing aimed to serve as a reference point, reinforcing the experience of naturalistic musical interactions (Rabinowitch et al., 2013).

Participants completed nine trials in total. After three trials, leadership roles were assigned for the remaining six trials to explore H3 (Table 2). When acting as a leader, participants were instructed to sing and initiate the clave performance, while followers were required to copy the leader’s performance without singing. Leadership roles were alternated between participants, with each participant completing three trials per role. The experiment lasted approximately 10 min. Six pairs were video recorded for transparency purposes.

### 2.6 Data processing and analysis

Onsets of the audio recordings were extracted in Python (version 3.7.7) using *Librosa* (version 0.8.1, McFee et al., 2015), relying on peak-picking in the onset strength envelope. A measure of synchrony between the extracted beats was calculated using the *onsetsync* R package (version 0.5.1; Eerola and Clayton, 2024). More precisely, we calculated absolute asynchrony between the participants’ onsets within pairs that were no more than 100 milliseconds (ms) apart. Utilizing absolute values was inspired by the study we replicated here (Novembre et al., 2019), avoiding situations in which keeping the sign (non-absolute asynchrony) would average to 0 ms when participants randomly switch positions in terms of leading or lagging. Additionally, we established the strength of the periodicity of each participant’s tapping by taking the peak amplitude of autocorrelations of trials that were phase-shifted between 0.2 and 1 s. This lag size reflected the likely outer range of tapping periodicity and was utilized to understand how stable each child’s tapping was during each trial of the experiment. The values were normalized prior to analysis by dividing each by the maximum value to facilitate interpretation of the results.

To assess participants’ rhythmic abilities, we estimated their tapping accuracy during the baseline task when they tapped along with the metronome. Synchronization accuracy was defined as the absolute asynchrony between the metronome and their tapping using the same procedure described above. For the part of the baseline task when the metronome had faded out, we calculated individual consistency of the continuation accuracy by taking the consistency of the tapped periods by calculating the coefficient of variation for the onset time differences between the successive taps.

To investigate the effects of trait empathy and the assignment of leadership roles on children’s interpersonal synchrony (H1-H3), linear mixed-effects models (LME) were run using the package lme4 (Bates et al., 2020) in RStudio (RStudio Team, 2020). The models utilized “absolute asynchrony” as the main dependent variable, indicating pairs’ average asynchrony for each trial. Therefore, high values of this variable would indicate low levels of interpersonal synchrony. Trait empathy and its three facets were treated as continuous variables comprising total scores of participants’ ratings on CASES. Leadership assignment was considered a three-level factor, delineating trials where a participant was a leader, a follower or when no roles were assigned. We also examined the effects of individuals’ temporal regularity, as well as individuals’ rhythmic accuracy and consistency, as evaluated in the baseline task. Figure 2 presents a summary of all variables used in the models. All assumptions (i.e., normality of residuals, linearity between predictors and response variable and homoscedasticity) were satisfied, and the diagnostic tests conducted are reported in Supplementary Appendix 4.

To identify the most influential random effects, null models with no fixed effects were initially run, including random intercepts for schools, trials, pairs and participants and intercepts for pairs varying within schools and participants within pairs (Bousquet, 2021). Subsequently, the models were gradually simplified, removing random effects explaining close-to-zero variance. Where variance was not zero, each model was compared with a reduced one by assessing the Akaike Information Criterion (AIC), chi-square estimates, and associated *p*-values via the ANOVA function in RStudio (using Restricted Maximum Likelihood - REML). In cases where models did not demonstrate significant differences, the model with the smallest AIC value was selected, favoring a simpler structure.

A hypothesis-driven minimal approach was then applied to investigate the fixed effects of trait empathy and leadership on absolute asynchrony. Starting with trait empathy as a total score, we gradually added more predictors to the model, including main effects and interactions between leadership assignment and each empathic facet, aligning with the research questions. We further added individuals’ temporal regularity and their rhythmic accuracy and consistency into the models to investigate their potential main effects and interactions with empathy and leadership roles. Using the ANOVA function and Maximum Likelihood (ML), each new model was compared with its preceding one, and predictors not significantly improving the fit of the model were removed (Schmidt et al., 2016). Significance was assessed based on *p*-values obtained from the *lmerTest* R package (Kuznetsova et al., 2017) using the Satterthwaite (1946) approximation. When model comparisons did not indicate a significant difference (*p* > 0.05), the simpler model with a lower AIC was chosen.

For exploratory purposes, we examined whether familiarity within pairs, participants’ gender and prior musical experiences, as well as pairs’ gender composition (female–female, male-make or mixed) had any confounding effects on children’s interpersonal synchrony. These factors were tested considering their previously observed influence on synchrony and empathy (Timmers et al., 2020; Gaggioli et al., 2019; Fujiwara et al., 2019; Schulte-Rüther et al., 2008). The R packages *emmeans* (Lenth, 2021) and *ggplots2* (Wickham, 2016) were utilized to perform post-hoc comparisons (using Tukey’s adjustment) and visualize the identified effects, respectively.

## 3 Results for Experiment 1: “Free synchrony task”

The final model of pairs’ absolute asynchrony indicated a significant interaction between individuals’ trait empathy (total score) and temporal regularity, as well as a significant main effect of pairs’ gender composition (Table 4). The inclusion of leadership roles and individuals’ rhythmic accuracy and consistency from the baseline task did not improve the fit of the model.

**Table 5:** Pairwise comparisons between levels of pairs’ gender composition in the model of absolute asynchrony using Tukey’s adjustment for multiple comparisons.

Pairwise Comparisons	*β*	*SE*	*df*	*t*	*p*
**Mixed pairs - Female/Female pairs**	**3.38**	**1.16**	**144**	**2.91**	0.011*
Mixed pairs - Male/Male pairs	−1.63	1.31	143	−1.24	0.429
**Female/Female pairs - Male/Male pairs**	**−5.01**	**1.45**	**144**	**−3.44**	0.002**

*β*: Coefficient estimate, *SE*: Standard Error*; df:* Degrees of freedom; *t*-values and *p*-values associated with *β.* The values in bold indicate a significant effect. Significance levels: ***p < 0.001, **p < 0.01, *p < 0.05.

**Figure 3:**
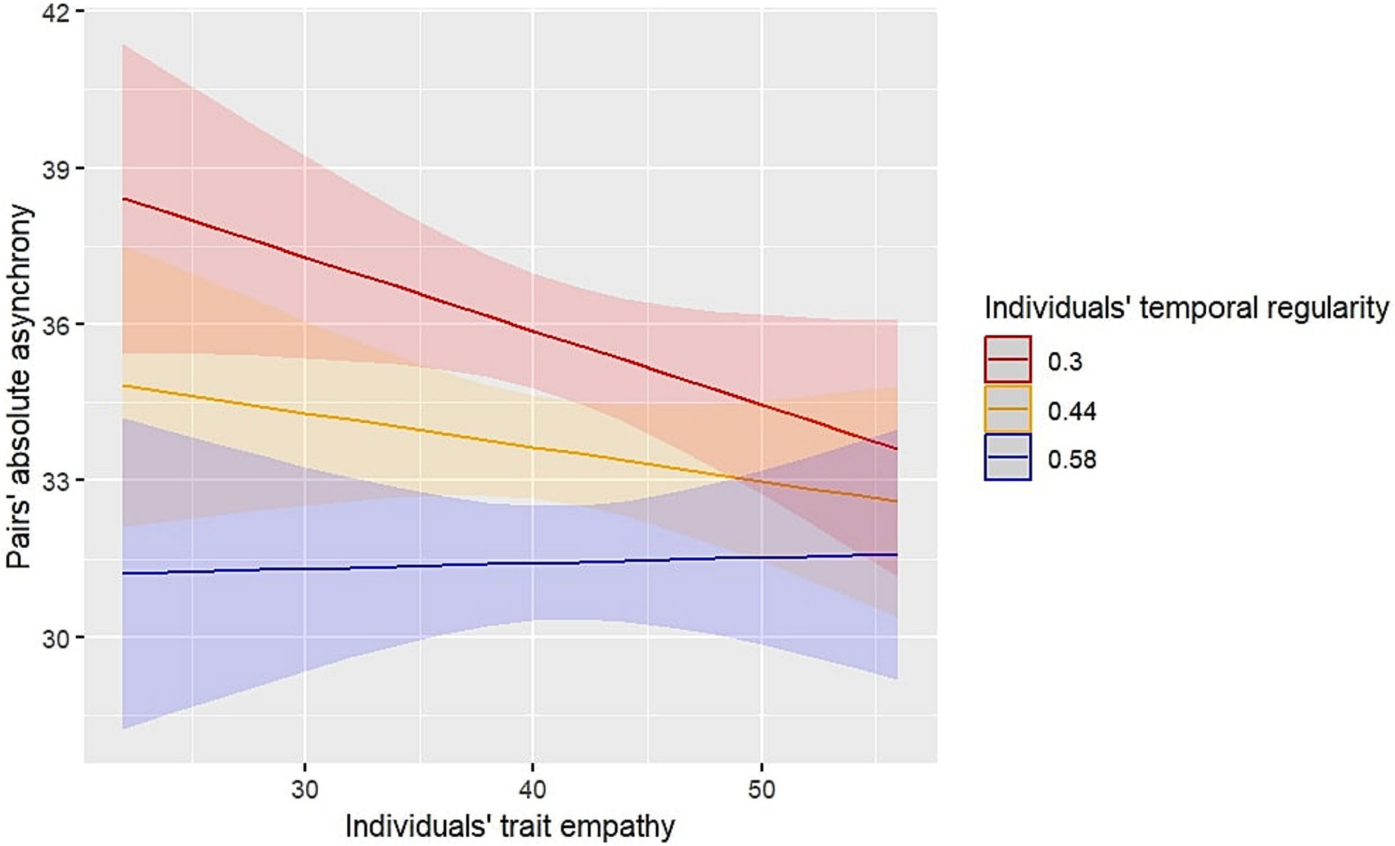
Predicted values of absolute asynchrony within pairs. Pairs’ absolute asynchrony is in milliseconds (ms). The shaded areas represent 95% Confidence Intervals. Lower values of absolute asynchrony indicate better synchrony within pairs. Higher temporal regularity indicates more stable participants’ tapping.

Focusing on the significant interaction between trait empathy and temporal regularity, Figure 3 indicates that higher empathy of individuals (total score) was associated with improved synchrony when children’s tapping was more irregular. Looking at the effect of empathy across three levels of individuals’ temporal regularity (high, moderate and low), children with high empathy were more synchronized than those with low empathy in trials when individuals’ tapping was unstable. However, for those with strong internal periodicity (more regular tapping), the effect of empathy is not evident. In other words, increased empathy was associated with better interpersonal synchrony, particularly in trials where individuals’ temporal regularity was low or moderate.

Furthermore, we sought to explore which empathic facet (cognitive, affective or somatic) contributed more to the observed interaction between empathy and temporal regularity; therefore, we ran separate models with each empathic manifestation and reported their parameter estimates in Supplementary Appendix 5. Only the model of cognitive empathy indicated a significant interaction between individuals’ empathy and temporal regularity on pairs’ absolute asynchrony [*β* = 1.62, *SE* = 0.52, *t*(1239.75) = 3.09, *p* = 0.002], while both the cognitive and affective models demonstrated a main effect of individuals’ empathy on pairs’ absolute asynchrony [Cognitive empathy: *β* = −0.71, *SE* = 0.27, *t*(674.29) = −2.54, *p* = 0.011; Affective empathy: *β* = −0.69, *SE* = 0.29, *t*(658.07) = −2.36, *p* = 0.018]. The negative estimates (*β*) of these effects suggest that higher levels of cognitive and affective empathy were associated with lower levels of absolute asynchrony, thus better interpersonal synchrony for highly empathic children. Somatic empathy was not found to significantly influence absolute asynchrony here.

**Figure 4:**
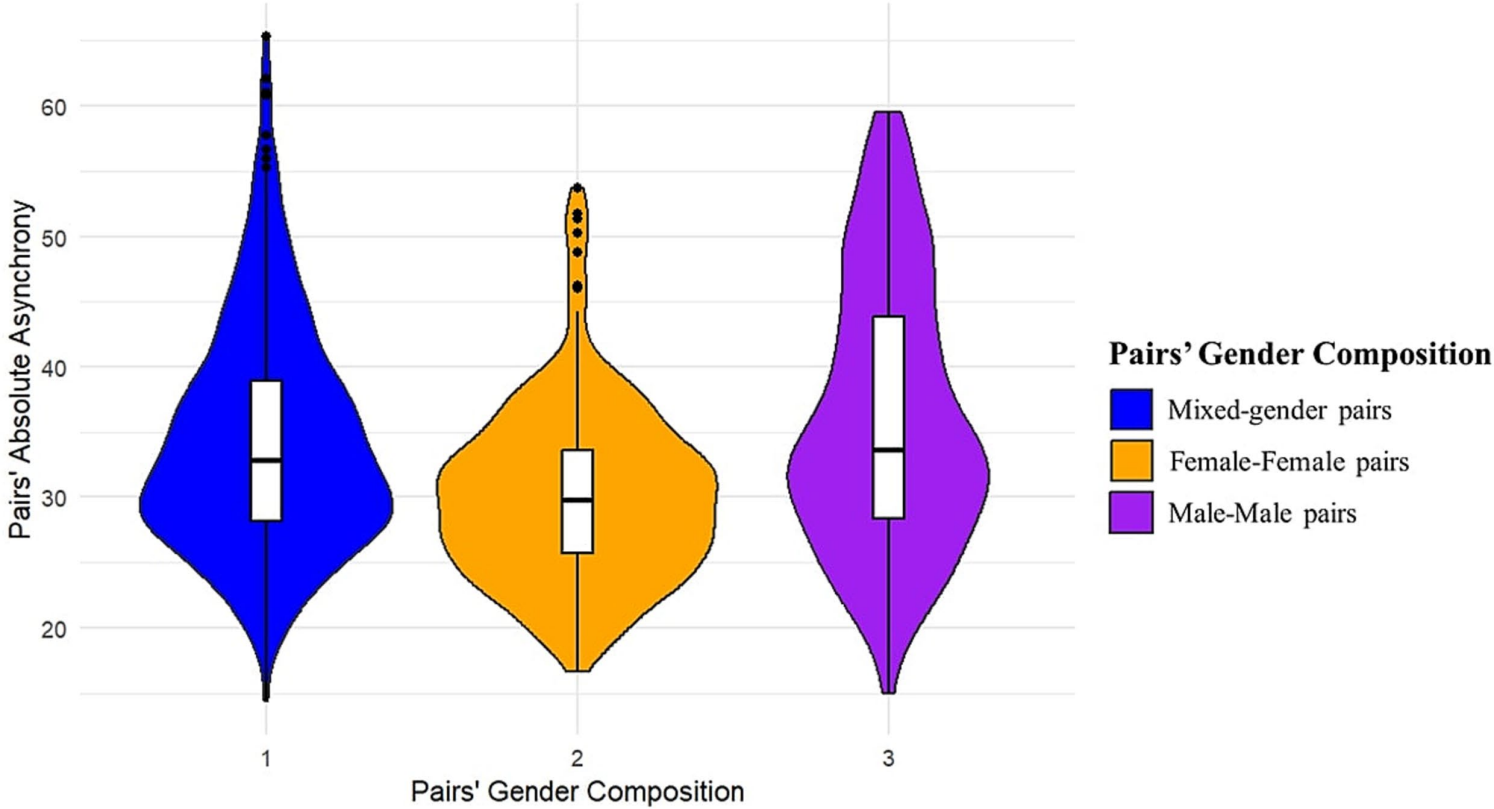
Distribution of pairs’ absolute asynchrony across the three levels of pairs’ gender composition. The width of each violin corresponds to the density of the data at different values of pairs’ absolute asynchrony (in ms). The line in the middle of the boxplots represents the median. Lower pairs’ absolute asynchrony indicated better synchrony.

Turning now to the significant effect of pairs’ gender composition, post-hoc analyses compared the three levels of the variable, i.e., (a) mixed, (b) female–female, and (c) male–male pairs. The comparisons revealed that female–female pairs synchronized significantly better than male or mixed-gender pairs (Table 5; Figure 4). We also explored the confounding effects of familiarity within pairs, participants’ gender and prior musical experiences on pairs’ absolute asynchrony; however, none of these variables improved the final model.

## 4 Methods for Experiment 2: “Manipulated synchrony task”

### 4.1 Summary

Following Experiment 1, participant proceeded to the second experiment, engaging again in brief musical interactions. While the task required again to synchronize with a partner, half of the pairs were intentionally misled to create asynchronous interactions. This design enabled examining the effects of synchrony (compared to asynchrony) on social bonding and state empathy (H4). Using a pretest-posttest approach, pairs’ feelings of social bonding and state empathy toward one another were assessed through self-reported questionnaires before and after the musical task. Participants’ trait empathy was included in the analysis to address H5 and H6. Additionally, half of the pairs were exposed to an empathy-inducing message about their partner, allowing for the investigation of H7 and H8.

### 4.2 Participants

The same participants completed Experiment 2. The final sample was slightly different here as data from participants excluded from one experiment were included in the other where appropriate. In total, 138 children (69 pairs) with a mean age of 11.05 years (*SD* = 0.72) completed Experiment 2. Table 3 presents demographic information.

### 4.3 Questionnaires

A self-reported questionnaire assessed participants’ feelings of bonding with their musical partners. Specifically, the questionnaire (Table 6) assessed children’s feelings of (1) closeness, (2) perceived similarity with their partner, and (3) ability to empathize with them (state empathy). These specific facets of social bonding were selected due to their relation with interpersonal synchrony observed in previous studies (Tzanaki et al., 2024; Koehne et al., 2016; Rabinowitch and Knafo-Noam, 2015). The questionnaire was administered twice, first before and then after the musical interactions of the experiment. The order of questions was randomized for the second round to minimize the influence of participants’ memory on their responses.

**Table 6:** The social bonding questionnaire and Cronbach’s alpha coefficients assessing internal consistency.

Questions	Social bonding aspect	Pre-test Cronbach’s *α*	Post-test Cronbach’s *α*
1. Inclusion of Other in the Self (IOS): Figure 5	Closeness	0.63 *(for questions 1–8, assessing social bonding)*	0.72 *(for questions 1–8, assessing social bonding)*
2. Do you think the other pupil has the same hobbies as you do?	Perceived similarity		
3. Do you think they like the same type of music as you?			
4. Do you think the other pupil is similar to you in character?			
5. Do you think you can guess the other pupil’s thoughts?	State empathy	0.51 *(for questions 5–8, assessing state empathy)*	0.62 *(for questions 5–8, assessing state empathy)*
6. Do you think you can understand how the other pupil is feeling at the moment?			
7. If you saw the other pupil happy, would that make you feel happy?			
8. If you saw the other pupil sad, would that make you feel sad?			

Values greater than 0.5 demonstrate acceptable internal consistency, given that the questionnaire contains less than 10 items (Pallant, 2013).

**Figure 5:**
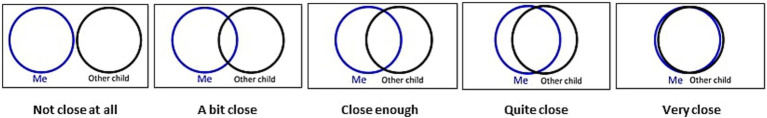
The Inclusion of Other in Self (IOS) Scale (Aron et al., 1992) as adapted for the present study.

Looking into each social bonding facet separately, closeness was evaluated using the Inclusion of Other in Self (IOS) scale (Aron et al., 1992) as adapted for primary school children by Rabinowitch and Knafo-Noam (2015). Participants were introduced to five sets of circles (Figure 5) representing gradual levels of closeness between themselves (black circle) and their partner (blue circle). Children were individually asked to choose the set of circles that best illustrated how close they felt to their partner. Descriptive phrases accompanied the circles to facilitate understanding.

Perceived similarity, the process of observing common qualities, abilities and values with another individual (Graves and Elsass, 2005), was assessed via Questions 2–4 (Table 6), as used by Rabinowitch and Knafo-Noam (2015). The questions focused on children’s character and musical preferences and were rated on a 3-point Likert scale (*“Yes,” “Maybe,” “No”*). Finally, Questions 5–8 (Table 6), obtained from Koehne et al. (2016), measured children’s ability to empathize with their partners (state empathy). As these questions had not been previously used with children of this age, they underwent review by two primary school teachers and were tested in a pilot study, confirming their comprehension and suitability for the intended age group. The state empathy questions were answered on a 3-point Likert scale, as with perceived similarity.

Two composite social bonding scores for each participant were computed as the total score of their ratings provided before and after Experiment 2 (see Table 6 for Cronbach’s *α* coefficients). Additionally, separate state empathy scores were calculated for each child to explore the effects of synchrony on this particular affiliative aspect.

### 4.4 Stimuli and equipment

Experiment 2 required manipulating the level of synchrony within pairs to examine its effects on social bonding and state empathy. Using Audio-Technica ATH-M20x headphones, participants listened to excerpts of a Greek children’s song (see Supplementary Appendix 2 for details) while instructed to play on the beat of the music using percussion instruments. Six bars of a metronome, two before the song began and four more into the music, aimed to help children identify the beat. Children were offered a selection of claves, tambourines, wooden scrapers and maracas to choose from for this experiment.

Pairs were randomly allocated to either the synchronous or asynchronous condition, both involving four 30-s-long trials. In the synchronous condition, all trials presented the song at 120 bpm for both children, whereas in the asynchronous condition, a slower version (90 bpm) was presented to one of the participants, alternating in every trial. The musical stimuli were created and produced in Audacity (3.2.1).

To induce empathy for this experiment, half of the pairs listened via their headphones to a 30-s-long message explaining that their musical partners lost their favorite toy/game on that day and encouraging them to imagine their emotional state. This message was delivered in Greek, and a translated version into English can be found in Supplementary Appendix 3. Prior to the study, the message was discussed with two primary school teachers who confirmed its appropriateness for this age group.

### 4.5 Experimental procedure

Participants completed the pre-test social bonding questionnaire, whereafter they chose a percussion instrument for the task. The experiment commenced with a practice trial, in which children listened to the experiment song at 120 bpm and performed individually on the beat of the music using their chosen percussion instrument. Unbeknown to participants, pairs were allocated to either the synchronous or the asynchronous condition, and half of the pairs of each condition were also allocated to the empathy manipulation group (Table 7), listening to the empathy message before the experiment. The experimenter, who remained present, was not blinded to the conditions, as simple observation of children’s performance could reveal their experimental condition. Nonetheless, they remained silent, avoiding eye contact with the participants.

**Table 7:** Distribution of synchrony and empathy manipulation conditions across pairs.

Groups	Synchrony conditions	Empathy manipulation	Distribution
1	Synchronous (i.e., same tempo of background music)	Yes	19 pairs
2	Synchronous (i.e., same tempo of background music)	No	16 pairs
3	Asynchronous (i.e., different tempo of background music)	Yes	17 pairs
4	Asynchronous (i.e., different tempo of background music)	No	17 pairs

During the experiment, participants were instructed to imagine performing in a music band and to play on the beat of the music while facing each other. Their strokes were not recorded this time to facilitate the use of a wider range of instruments. Following the four trials of the experiment, participants completed the social bonding questionnaire again (post-test) and were debriefed before returning to their classroom. The experiment lasted approximately 10 min. Again, six pairs were video recorded for transparency purposes.

### 4.6 Data processing and analysis

The linearity and homoscedasticity assumptions for LME were not met here due to the ordinal nature of the response variables. Therefore, cumulative mixed-effects models (CLMMs) were run instead via the *ordinal* package in RStudio (Christensen, 2018). To further satisfy the assumptions for these models, the social bonding and state empathy ratings were transformed into ordinal variables by creating four ranges (0 for scores <0.5, 1 for scores 0.5–1, 2 for scores 1–1.5, 3 for scores 1.5–2).

Two separate models were run, one for social bonding and one for state empathy. Both models investigated the effects of synchrony (synchrony or asynchrony), induced empathy (empathy manipulation applied or not), trait empathy (total score and separate empathic facets), time (pre- and post-test ratings) and their interactions on children’s social bonding (average of closeness, perceived similarity and state empathy scores) and state empathy ratings, separately. Figure 2 presents a summary of all variables used in the models.

To identify the random effects of the two models, a similar approach to Experiment 1 was first followed. A hypothesis-driven minimal approach was then applied, investigating the effects of the four predictors on social bonding and state empathy. For both models (social bonding and state empathy models), we started with structures containing only “time,” assessing changes in the response variables post-experiment. We gradually added more predictors, keeping only those that significantly improved the fit of the models. Finally, the impact of familiarity within pairs, participants’ gender, pairs’ gender composition and prior musical experiences were also examined here, exploring their confounding effects on social bonding and state empathy.

## 5 Results for Experiment 2: “Manipulated synchrony task”

**Figure 6:**
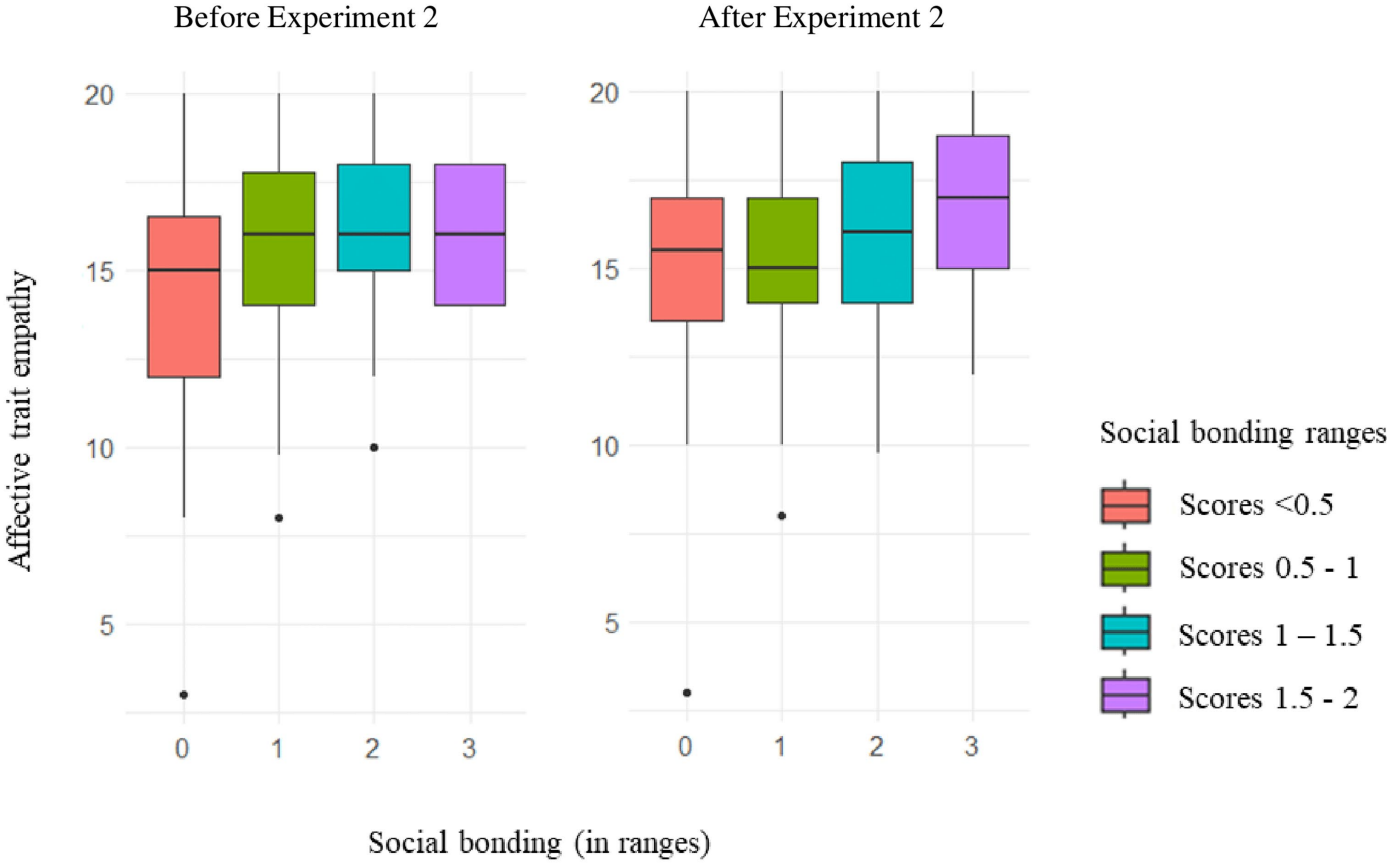
The effects of affective trait empathy on social bonding before and after Experiment 2. The size of the boxes represents the interquartile range (IQR), the range containing the middle 50% of the data. The line in the middle of the boxes indicates the median of the distribution. The social bonding variable was transformed into an ordinal variable with four ranges, as indicated by the separate boxes.

Table 8 presents the final CLMM models for social bonding and state empathy. Starting with the social bonding model, only the main effects of time and affective empathy remained in the final model. The effects of synchrony and empathy manipulation did not improve the fit of the model and were, therefore, excluded. The effect of time indicated that all participants experienced higher social bonding following their musical interaction than before it (*β* = −1.98, *SE* = 0.35, *z* = −5.59, *p* < 0.001), regardless of the synchrony and empathy manipulation conditions assigned. Regarding affective empathy, those with higher affective empathy tended to report higher social bonding with their partners than those with lower affective empathy in both questionnaires, as illustrated in Figure 6 (*β* = 0.29, *SE* = 0.10, *z* = 2.91, *p* = 0.003).

**Table 8:** The best-fitting CLMM models for (a) social bonding and (b) state empathy ratings.

Dependent variable	Fixed effects	Random effects	AIC	BIC
(a) Social bonding	Time + Individuals’ affective empathy + Familiarity + Pairs’ gender composition	(1| Pair) + (1| Participant)	553.43	600.49
Fixed effects	*β*	*SE*	*z*	*p*
**Time 2 (post-test)**	**1.94**	**0.35**	**5.46**	<0.001***
**Individuals’ affective empathy**	**0.31**	**0.10**	**3.09**	0.001**
Familiarity level 2: *“We know each other a little bit”*	1.00	0.66	1.50	0.133
**Familiarity level 3:** ***“We know each other quite a bit”***	**2.56**	**0.90**	**2.84**	0.004**
**Familiarity level 4:** ***“We know each other well”***	**4.73**	**1.43**	**3.30**	<0.001***
**Familiarity level 5: *“We know each other very well/we are friends”***	**7.69**	**1.99**	**3.86**	<0.001***
**Female–female pairs (F-F)**	**2.35**	**0.74**	**3.17**	0.001**
Male–male pairs (M-M)	0.33	0.80	0.41	0.680
Dependent variable	Fixed effects	Random effects	AIC	BIC
(b) State empathy	Time * Synchrony + Individuals’ trait empathy (total score) + Familiarity	(1| Participant)	625.01	668.46
Fixed effects	*β*	*SE*	*z*	*p*
Time 2 (post-test)	0.25	0.38	0.66	0.503
Synchrony 1 (Synchronous condition)	−0.52	0.61	−0.85	0.392
**Individuals’ trait empathy** **(total score)**	**0.15**	**0.04**	**3.73**	<0.001***
Familiarity level 2: *“We know each other a little bit”*	0.75	0.64	1.16	0.242
**Familiarity level 3:** ***“We know each other quite a bit”***	**2.20**	**0.83**	**2.63**	0.008**
**Familiarity level 4:** ***“We know each other well”***	**3.12**	**1.31**	**2.37**	0.017*
**Familiarity level 5: *“We know each other very well/we are friends”***	**4.60**	**1.58**	**2.89**	0.003**
Time 2 * Synchrony 1	**1.51**	**0.56**	**2.69**	0.006**

The social bonding model allowed for random intercepts for pairs and participants, and the state empathy model allowed for random intercepts for participants. *β*: Coefficient estimate, *SE*: Standard Error*; z*-values and *p*-values associated with *β.* The values in bold indicate a significant effect. Significance levels: ***p < 0.001, **p < 0.01, *p < 0.05. RStudio compared the levels of the predictors below with the reference levels: Time 1: pre-test; Familiarity within pairs 1: “*We do not know each other at all”*; Pairs’ gender composition 1: Mixed pairs; Synchrony 0: Asynchronous condition.

Furthermore, for exploratory purposes, we examined the potential confounding effects of previous acquaintance (familiarity) within pairs, the role of gender and its interaction with affective trait empathy, as well as participants’ previous musical experience. Previous acquaintance improved the fit of the model, indicating that greater familiarity between participants was associated with higher social bonding ratings, regardless of the musical interaction, synchrony and empathy manipulation conditions. The coefficient estimate for the contrast between level 1 (*“Not knowing each other at all”*) and level 5 (*“Knowing each other very well/we are friends”*) was −7.80. (*SE* = 2.01, *z* = −3.84, *p* = 0.001), indicating significantly higher levels of social bonding for participants who knew each other very well. Additionally, we examined whether the pairs’ gender composition presented any differences, a factor that improved the fit of the final social bonding model (Table 8). Indeed, female–female pairs tended to report significantly higher social bonding than male–male or mixed pairs (Contrast between mixed and female–female pairs: *β* = −2.33, *SE* = 0.73, *z* = −3.18, *p* = 0.004; contrast between female–female and male–male pairs: *β* = 2.14, *SE* = 0.86, *z* = 2.47, *p* = 0.035).

**Figure 7:**
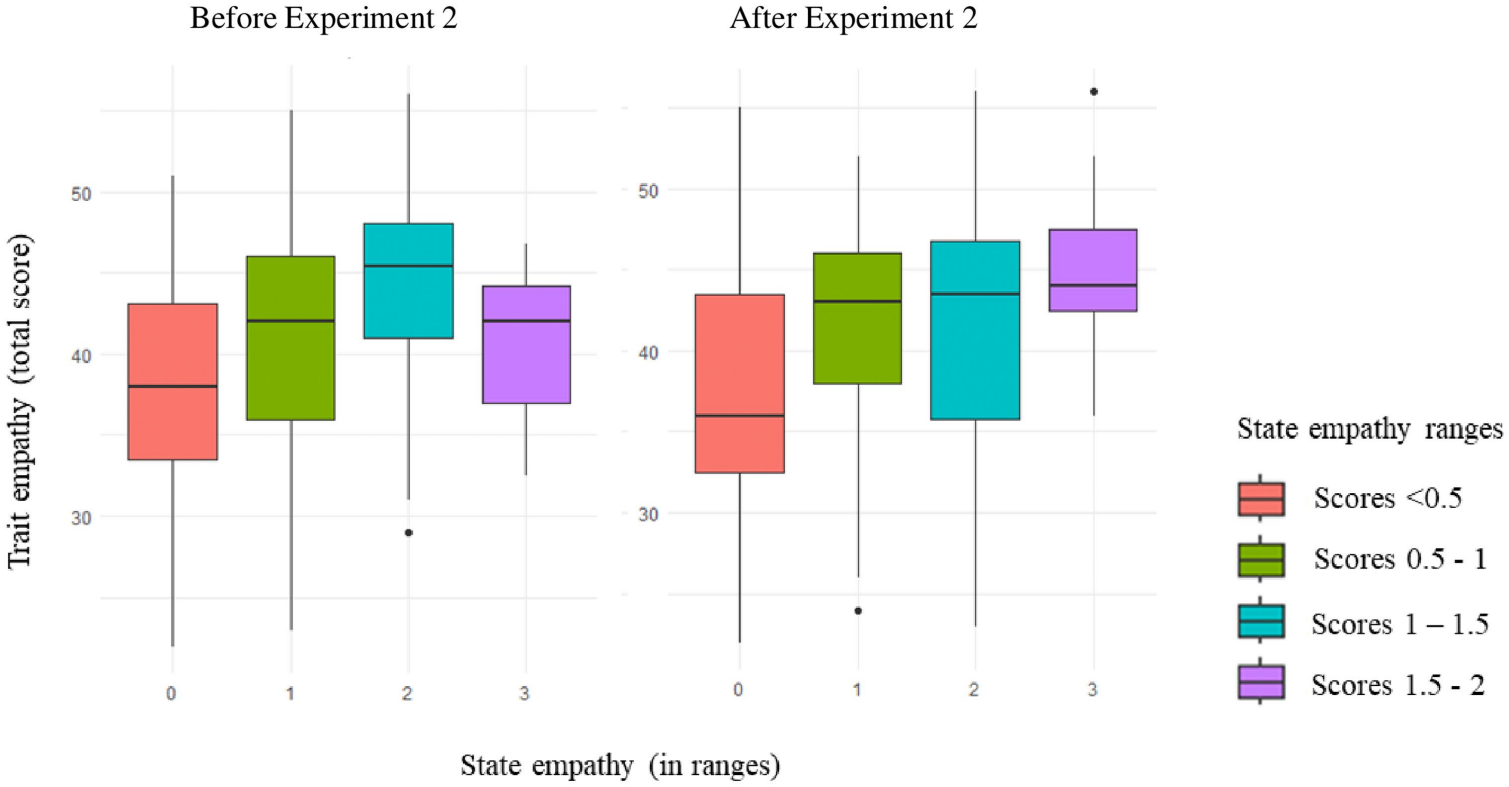
The effects of trait empathy (total score) on state empathy before and after Experiment 2. The size of the boxes represents the interquartile range (IQR), the range containing the middle 50% of the data. The line in the middle of the boxes indicates the median of the distribution. The social bonding variable was transformed into an ordinal variable with four ranges, as indicated by the separate boxes.

Focusing now on the best-fitting model for state empathy (Table 8), a significant interaction between time and synchrony indicated that participants in the synchronous conditions reported higher state empathy ratings following Experiment 2 than before it (*β* = −1.78, *SE* = 0.40, *z* = −4.39, *p* < 0.001). This was not the case for participants in the asynchronous conditions, whose scores did not vary significantly between the pre- and post-test measurements (*p = 0*.837). In addition to these effects, trait empathy as a total score significantly improved the fit of the model, confirming, as expected, that participants with higher trait empathy tended to provide higher state empathy scores than those with lower empathy (*β* = 0.14, *SE* = 0.03, *z* = 3.65, *p* < 0.001). Figure 7 illustrates the effects of trait empathy on the pre- and post-test state empathy ratings. Induced empathy did not improve the fit of the model.

Finally, as in the social bonding model, we explored the effects of the confounding variables of interest and found that previous acquaintance improved the fit of the state empathy model, associating greater familiarity with the other child with increased state empathy. The coefficient estimate of state empathy for the contrast between level 1 (*“Not knowing each other at all”*) and level 5 (*“Knowing each other very well / we are friends”*) was −4.55. (*SE* = 1.59, *z* = −2.86, *p* = 0.03). The effects of participants’ gender, pairs’ gender composition and prior musical experiences were tested without improving the fit of the final model.

## 6 General discussion

This study investigated three aspects of the relationship between empathy and synchrony in children’s musical interactions, providing empirical evidence for theoretical claims of the feedback loop model (Tzanaki, 2022). Experiment 1 tested the direction from empathy to synchrony, namely the role of trait empathy in facilitating children’s capacity to synchronize with one another. The reverse direction was examined in Experiment 2, testing whether synchronizing briefly with others can encourage social bonding and state empathy and whether these effects are modulated by trait or induced empathy. For exploratory purposes, we investigated the confounding effects of familiarity within pairs, their gender, prior musical experiences, and pairs’ gender composition. This was motivated by previous findings suggesting an impact of such factors on synchrony and empathy (Timmers et al., 2020; Gaggioli et al., 2019; Fujiwara et al., 2019; Schulte-Rüther et al., 2008).

Our results support the first hypothesis (Table 2), revealing that children with higher trait empathy synchronized better than those with lower empathy. However, this was prominent only when children’s tapping was less stable, suggesting that where participants’ temporal behavior was more irregular, synchrony benefited from children with high empathy. This could be attributed to empathy supporting the capacity to predict others’ temporal behavior, as previously suggested by Novembre et al. (2019). We speculate that highly empathic children synchronized better as they could anticipate their partners’ actions more effectively when periodicity was less regular. Conversely, for trials with more consistent tapping, synchronizing did not require superior predictive skills, leading to relatively good alignment without the support of individuals’ empathy.

Looking at each empathic facet, we observed that in addition to cognitive empathy (as also seen in Novembre et al., 2019), higher affective empathy was also associated with enhanced interpersonal synchrony. Relevant research supports that top-down empathic processes, such as consciously adopting others’ perspectives, rely on an automatic emotional alignment (de Waal, 2008). Therefore, it is plausible that playing music in Experiment 1 facilitated emotional alignment (Cross et al., 2012), activating automatic affective sharing. This might have resulted in children with higher cognitive and affective empathy utilizing their predictive skills (Novembre et al., 2019) and the emotional alignment stemming from this shared experience to a greater extent, thereby synchronizing better. Nonetheless, it is important to highlight here that although our analysis revealed important associations between empathy and synchrony, more work is required to prove causal effects. For example, future research could explore the developmental trajectories of empathy and synchrony, investigating to what extent one process shapes the other (Feldman, 2007), and the role of music in this context. Additionally, it would be valuable to investigate whether other factors, such as keen perceptiveness, contribute to the development of both synchrony and empathy in such interactions.

The model in Experiment 1 did not indicate a significant interaction between empathy and leadership roles, as observed by Novembre et al. (2019). This might be due to the limited number of trials per role and the frequent role change. Otherwise, our exploratory analysis showed that dyads of female participants synchronized better than male–male and mixed-gender pairs. This is consistent with prior research indicating that females present more synchronous behavior in social interactions than male participants (Fujiwara et al., 2019; Paolizzi et al., 2022). Pairs’ gender composition was also a significant predictor in the social bonding model of Experiment 2 (Table 8), demonstrating that female pairs typically reported stronger bonding with their partners than the other pairs. These gender-related findings collectively reflect the social dynamics inherent in musical interactions, corroborating previously observed gender differences in the development of coordination and interpersonal skills (Pahlevanian and Ahmadizadeh, 2014; Hajovsky et al., 2022).

It is important to acknowledge that while the mixed-effect model above with random intercepts for participants nested within pairs aimed to account for the dependencies in the dependent variable (absolute asynchrony measured at pair level with empathy and temporal regularity assessed at participant level), this presents certain limitations. Specifically, while this approach allowed us to examine how individual-level variables contribute to the shared outcome of absolute asynchrony, it may not fully capture the asymmetric contributions of individuals within pairs. To further validate our findings, we constructed an additional mixed-effect model using pairs’ average empathy and temporal regularity, instead of the original individual-level predictors. The results revealed consistent patterns (see section 6 and Figure 8 in Supplementary Appendix) lending robustness to our original findings. However, future research would benefit from adopting alternative approaches to better disentangle individual contributions to pair-level outcomes. Furthermore, our *a priori* power analysis was based on the individual-level variables, consistent with our approach. Future studies focusing on pair-level variables would require larger samples sizes to ensure adequate power. Nonetheless, our findings, based on 72 pairs, offer valuable insights and serve as a solid foundation for future research with larger samples.

Turning to the reverse direction of the synchrony-empathy relationship, our analysis showed that the synchrony and empathy manipulations did not differentiate children’s social bonding ratings, as hypothesized (H4a and H7). Instead, all participants increased their ratings post-experiment, potentially due to them interacting musically with one another. However, a significant interaction between time and synchrony in the state empathy model indicated that the increase in these ratings post-experiment was predominantly driven by the synchronous condition. This aligns with Hypothesis 4b, suggesting that synchrony enabled participants to empathize more with their partners than those in the asynchronous condition.

A potential explanation for the synchrony manipulation not differentiating social bonding across conditions might lie in the modality via which asynchrony was perceived. Perceiving partners’ performance visually might not have been sufficient to create a substantial distinction between synchronous and asynchronous partners. Indeed, prior studies (e.g., Rabinowitch and Knafo-Noam, 2015) exposed their participants to synchrony conditions via auditory cues, and individuals tend to be more sensitive to timing discrepancies via auditory than visual cues (Iversen et al., 2015). Therefore, the visually perceived asynchrony here might not have been adequate to diminish the positive effects of the shared musical task (Cross et al., 2012). Nonetheless, that was not the case for state empathy; our observation aligns with outcomes from studies in adults, indicating that synchrony encourages metalizing and a sense of understanding (Baimel et al., 2018; Koehne et al., 2016). It is possible that the synchronous condition created a channel of non-verbal communication (Wheatley et al., 2012), prompting participants to pay attention to their partners’ actions and fostering mentalizing and state empathy (Macrae et al., 2008; Baimel et al., 2018). Conversely, asynchronous interactions may have directed children’s attention to their own performance, disrupting communication and shared understanding.

The results above suggest that individuals from a young age appraise their partners during music-making, a process consequently influencing the social outcomes of synchrony and musical engagement (Lamm et al., 2007; Cross et al., 2012; Tzanaki et al., 2024). This is reinforced by our observation that participants who interacted with familiar partners provided higher social bonding and state empathy scores than those with no previous acquaintances. Given that social familiarity encourages state empathy (Preston and de Waal, 2002), our findings indicate that children utilized synchrony and familiarity with a partner as social cues to direct state empathy.

Finally, empathy induction was not a significant predictor in Experiment 2, rejecting Hypotheses 7 and 8. To minimize participant fatigue, we did not implement measures to assess the efficacy of the empathy message. Consequently, our method to induce empathy may not have been effective, possibly due to its short duration or because children were distracted by the musical interactions. Similar methods have been previously used in studies with adults (Van Lange, 2008; Miu and Baltes, 2012). However, the absence of prior validation of our manipulation with children remains a limitation of this study. Nonetheless, it highlights a valuable area for future research, where more effective methods could be explored, particularly for this age group. Furthermore, we did not observe a significant interaction between trait empathy and synchrony, to support that empathy influences the social bonding effects of interpersonal synchrony, as previously observed in adults (H5 and H6). Nonetheless, higher trait empathy was associated with stronger affiliation across all conditions, aligning with research linking trait empathy with prosocial behavior and the situational manifestations of empathy (Eisenberg et al., 2010; de Vignemont and Singer, 2006). Further work is required to examine whether longer musical interactions would allow children’s trait empathy to influence their appraisal processes and strengthen the social outcomes of synchrony.

Although some hypotheses were not confirmed, our results have made important contributions to the positive feedback loop model (Tzanaki, 2022), shedding light on additional factors influencing the loop’s functioning. Referring back to Figure 1, the study has confirmed that (a) trait empathy contributes to children’s ability to synchronize with others (Aspect 1), especially when temporal regularity is low, and (b) attained synchrony might act as a social cue for children to direct empathy in a given situation (Aspect 2). Our approach to inducing empathy or the short duration of Experiment 2 might explain why we could not confirm our hypotheses for Aspect 3. Nonetheless, the observed direct impact of trait empathy on the experience of social connection through music underscores the social dynamics of collective musical engagement (Cross et al., 2012). The study has also highlighted the susceptibility of the feedback loop to inter-individual or contextual factors, as previously proposed by Tzanaki (2022). Indeed, the outcomes imply that the characteristics of individuals with whom one interacts in a musical context—here, a familiar person or someone of the same gender—can influence the loop’s functioning.

Our findings can guide future longitudinal investigations directly exploring the bidirectional relationship of synchrony and empathy in musical contexts. However, certain considerations should be noted following some limitations of this study. Firstly, we chose absolute asynchrony to assess interpersonal synchrony to align with Novembre et al. (2019) methodology. However, this might present inaccuracies in cases where asynchronies are consistent but preserve a certain absolute value (e.g., when some are consistently ahead of others). To check the validity of our choice, we calculated a relative absolute asynchrony variable by dividing absolute synchrony by individuals’ temporal regularity to control for tempo changes. This new dependent variable yielded similar results; therefore, we opted for using absolute asynchrony to facilitate outcome interpretation. Future investigations could also use the variance of asynchronies, i.e., the variance of differences in onset timing, for a more nuanced understanding of pairs’ synchrony. Furthermore, given the substantial lack of research into the effects of induced empathy in musical interactions in young populations, future studies could extend our methodology, employing additional paradigms, such as video clips, role-playing or longer narratives, to provide additional insights into the role of empathy in experiencing bonding (Stupacher et al., 2022; Tzanaki et al., 2024).

To conclude, the study informed important developmental aspects of the positive feedback loop model (Tzanaki, 2022), revealing that trait empathy supports children’s ability to synchronize in musical interactions when children’s temporal performance is unstable. In addition to the role of cognitive empathy (Novembre et al., 2019), we found that affective empathy also supports synchrony, reinforcing emotional alignment in musical interactions. Brief exposure to visually perceived asynchrony was not sufficient to outweigh the positive effects of musical interactions on children’s social bonding, while short-term synchrony provided social cues for children to empathize with their partners (state empathy). Pairs’ gender composition and familiarity were found to influence synchrony and its social effects, highlighting the social dynamics of musical engagement. Future investigations could build upon these outcomes to inform educational interventions for promoting children’s musical and social development.

## Data Availability

The datasets presented in this study can be found in online repositories. The names of the repository/repositories and accession number(s) can be found at: https://orda.shef.ac.uk/articles/dataset/Actions_and_feelings_in_sync_Exploring_the_reciprocal_relationship_between_synchrony_and_empathy_in_children_s_dyadic_musical_interactions_Datasets_and_stimuli/25382701/3.
