# Supplementary material for: Actions and feelings in sync: exploring the relationship between synchrony and empathy in children’s dyadic musical interactions
Source: Front Psychol. 2025 Apr 25;16:1467767. doi: 10.3389/fpsyg.2025.1467767 (PMC12063534; doi:10.3389/fpsyg.2025.1467767)

**Appendix**

## **Participant exclusion process**

Some participants were excluded from data analysis for the following reasons:

- **Seven** pairs were excluded from both experiments because one or both children of the pair did not understand aspects of the experiments or were very distracted during the study, as the experimenter observed.
- **Three** children left multiple statements in the CASES questionnaire unanswered; therefore, their pairs were excluded from both experiments.
- Additionally, **three** children and their pairs were excluded from Experiment 2 as they left multiple questions unanswered in the social bonding questionnaires.

## **Experiment 2 music excerpt**

Participants in Experiment 2 listened to the Greek song “Συννεφούλα” (Sinnefoula) by Dionysis Savvopoulos (Album: Fortigho, released in 1966).

## **Empathy manipulation message**

Participants in the induced empathy conditions listened to the following message in Greek via their headphones before proceeding to the experiment. Please note that the words “toy” and “game” are the same in Greek and are considered appropriate for this age group.

*“Before we start this activity, I want to tell you a little secret. Earlier today, the other child lost their favourite toy/game, and they couldn’t find it. They looked everywhere, but they couldn’t find it. Can you imagine how they might be feeling at the moment? Try to imagine how you would feel if you had lost your favourite toy/game.”*

## **Assumption tests**

The final LME models of Experiment 1 were subjected to tests for the assumptions of a) normality of residuals, b) linearity, and b) homoscedasticity. These tests involved assessing Quantile-Quantile (Q-Q) plots depicting residuals, plots showing the relationship between residuals and fitted values, and Scale-Location (Spread-Location) plots examining homoscedasticity. The analyses were conducted via the R package *lattice* (Sarkar, 2008) in R Studio (RStudio Team, 2020). As for the final CLMM models of Experiment 2, these were subject to the assumptions of a) independence of observations and b) proportional odds, ensuring that the relationships between predictors and response variables are consistent across the ordinal categories (Christensen, 2018; Schmidt, 2012). For the first assumption, intraclass correlation coefficients were calculated, quantifying the proportion of total variance due to between-cluster variation. The second assumption was assessed on the same models without random effects (CLM) as the “nominal_test” and “scale test” used are not currently available for CLMM (Mangiafico, 2016). The code utilised to conduct these tests can be found here: <https://doi.org/10.15131/shef.data.25382545.v2>.

## **Parameter estimates for separate models of absolute asynchrony**

The table presents the separate models of absolute asynchrony that were run with each empathic facet as a predictor (a) Cognitive empathy; b) Affective empathy; c) Somatic empathy). For “pairs’ gender composition”, RStudio compared Female-Female and Male-Male pairs with mixed-gender pairs. AIC: Akaike Information Criterion. BIC: Bayesian Information Criterion. *β*: Estimate. *SE*: Standard Error. *df:* Degrees of freedom. *t-*values and *p*-values associated with *β*. The values in **bold** indicate a significant effect. Significance levels highlighted in bold: *p*<.001***, *p*<.01**, *p*<.05*.

1. Cognitive empathy

| Model | | | Random effects | AIC | | BIC |
| --- | --- | --- | --- | --- | --- | --- |
| Pairs’ absolute asynchrony ~  Individuals’ cognitive empathy * Individuals’ temporal regularity + Pairs’ gender composition | | | (1 \| Pair : Participant) | | 8208.65 | 8249.65 |
| Fixed effects | *β* | *SE* | *df* | *t* | | *p* |
| **(intercept)** | **51.11** | **4.06** | **668.54** | **12.57** | | **<.001***** |
| **Individuals’ cognitive empathy** | **-0.71** | **0.27** | **674.29** | **-2.54** | | **.011*** |
| **Individuals’ temporal regularity** | **-38.75** | **7.63** | **1240.73** | **-5.07** | | **<.001***** |
| **Ind cogn. emp. *   Ind. temp. regul.** | **1.62** | **0.52** | **1239.75** | **3.09** | | **.002**** |
| **Female-Female pairs (F-F)** | **-3.45** | **1.14** | **133.17** | **-3.01** | | **.003**** |
| Male-Male pairs (M-M) | 1.85 | 1.29 | 132.13 | 1.43 | | .153 |

1. Affective Empathy

| Model | | | Random effects | AIC | BIC |
| --- | --- | --- | --- | --- | --- |
| Pairs’ absolute asynchrony ~  Individuals’ affective empathy * Individuals’ temporal regularity + Pairs’ gender composition | | | (1 \| Pair : Participant) | 8212.00 | 8253.00 |
| Fixed effects | *β* | *SE* | *df* | *t* | *p* |
| **(intercept)** | **51.87** | **4.64** | **628.21** | **11.15** | **<.001***** |
| **Individuals’ affective empathy** | **-0.69** | **0.29** | **658.07** | **-2.36** | **.018*** |
| **Individuals’ temporal regularity** | **-29.02** | **8.82** | **1232.04** | **-3.28** | **.001**** |
| Ind. aff. emp. * Ind. temp. regul. | 0.85 | 0.56 | 1233.78 | 1.52 | .128 |
| **Female-Female pairs (F-F)** | **-3.43** | **1.12** | **133.53** | **-3.06** | **.002**** |
| Male-Male pairs (M-M) | 1.46 | 1.27 | 133.04 | 1.15 | .252 |

1. Somatic Empathy

| Model | | | Random effects | AIC | BIC |
| --- | --- | --- | --- | --- | --- |
| Pairs’ absolute asynchrony ~  Individuals’ somatic empathy * Individuals’ temporal regularity + Pairs’ gender composition | | | (1 \| Pair : Participant) | 8216.11 | 8257.10 |
| Fixed effects | *β* | *SE* | *df* | *t* | *p* |
| **(intercept)** | **44.28** | **3.07** | **635.44** | **14.39** | **<.001***** |
| Individuals’ somatic empathy | -0.27 | 0.24 | 646.49 | -1.09 | .344 |
| **Individuals’ temporal regularity** | **-23.67** | **5.76** | **1241.26** | **-4.11** | **<.001***** |
| Ind. som. emp. * Ind. temp. regul. | 0.66 | 0.46 | 1236.89 | 1.43 | .212 |
| **Female-Female pairs (F-F)** | **-3.44** | **1.14** | **133.24** | **-3.01** | **.003**** |
| Male-Male pairs (M-M) | 1.83 | 1.29 | 132.84 | 1.41 | .218 |

## **Additional analysis for Experiment 1 with predictors aggregated at the pair level**

The model below was constructed to compare with the model in Table 4. The same predictors were used here but were aggregated at the pair level. For “pairs’ gender composition”, RStudio compared Female-Female and Male-Male pairs with mixed-gender pairs. AIC: Akaike Information Criterion. BIC: Bayesian Information Criterion*. β:* Coefficient estimate. *SE*: Standard Error. *df:* Degrees of freedom. *t*-values and *p*-values associated with *β. η_p_^2^:* Partial eta-squared measuring effect size: Small=.01; Medium=.06; Large=.14. CI: Confidence Intervals. Significance levels highlighted in bold: *p*<.001***, *p*<.01**, *p*<.05*. The significant interacting effect of pairs’ average empathy and temporal regularity on pairs’ absolute asynchrony is presented in Figure 8.

| Model | Random effects | AIC | BIC |
| --- | --- | --- | --- |
| Pairs’ absolute asynchrony ~  Pairs’ average empathy * Pairs’ average temporal regularity + Pairs’ gender composition | (1 \| Pair : Participant) | 8170.53 | 8211.52 |

| Fixed effects | *β* | *SE* | *df* | *t* | *p* | *η_p_^2^* | 95% CI |
| --- | --- | --- | --- | --- | --- | --- | --- |
| **(intercept)** | **63.64** | **8.55** | **704.67** | **7.44** | **<.001***** | **-** | **[46.88, 80.40]** |
| **Pairs’ average empathy** | **-0.46** | **0.20** | **708.63** | **-2.24** | **.025*** | **.007** | **[-0.86, -0.05]** |
| **Pairs’ average temporal regularity** | **-53.52** | **14.30** | **1074.58** | **-3.74** | **<.001***** | **.01** | **[-81.57, -25.48]** |
| **Pairs’ av. emp. *  Pairs’. av. temp. regul.** | **0.76** | **0.34** | **1067.71** | **2.23** | **.025*** | **.004** | **[0.09, 1.44]** |
| **Female-Female pairs (F-F)** | **-2.97** | **1.06** | **132.17** | **-2.78** | **.006**** | **.08** | **[-5.06 -0.87]** |
| Male-Male pairs (M-M) | 1.39 | 1.21 | 134.27 | 1.14 | .255 | .08 | [-0.99, 3.78] |

**Figure 8:**

The figure shows the significant interacting effect of pairs’ average empathy and temporal regularity on pair’s absolute asynchrony. This was created following an additional analysis for Experiment 1 with predictors aggregated at the pair level to further validate the original analysis (see General Discussion and Appendix). Pairs’ absolute asynchrony is in milliseconds (ms). The shaded areas represent 95% Confidence Intervals. Lower values of absolute asynchrony indicate better synchrony within pairs. Higher temporal regularity indicates more stable pairs’ tapping.


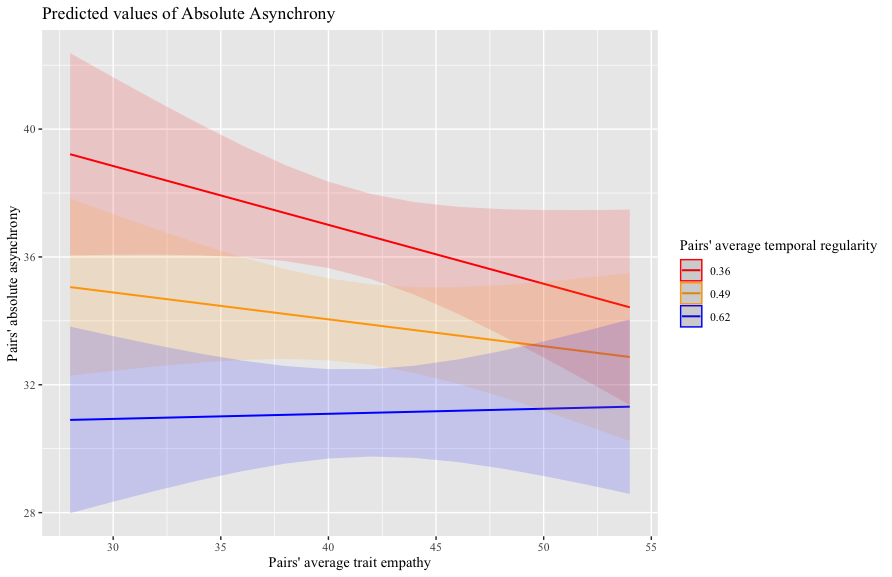

Supplement: Supplementary file 1 [file Supplementary_file_1.docx]
